# Electron-phonon coupling in topological surface states: The role of polar optical modes

**DOI:** 10.1038/s41598-017-01128-1

**Published:** 2017-04-24

**Authors:** Rolf Heid, Irina Yu. Sklyadneva, Evgueni V. Chulkov

**Affiliations:** 1 Institut für Festkörperphysik, Karlsruher Institut für Technologie, D-76021 Karlsruhe, Germany; 2 Donostia International Physics Center (DIPC), 20018 San Sebastián/Donostia, Basque Country, Spain; 3 St. Petersburg State University, 199034 St. Petersburg, Russian Federation; 4 Departamento de Física de Materiales, Facultad de Ciencias Químicas, UPV/EHU, Apdo. 1072, 20080 San Sebastián/Donostia Basque Country, Spain; 5 Centro de Física de Materiales CFM - Materials Physics Center MPC, Centro Mixto CSIC-UPV/EHU, 20018 San Sebastián/Donostia Basque Country, Spain

## Abstract

The use of topological edge states for spintronic applications could be severely hampered by limited lifetimes due to intrinsic many-body interactions, in particular electron-phonon coupling. Previous works to determine the intrinsic coupling strength did not provide a coherent answer. Here, the electron-phonon interaction in the metallic surface state of 3D topological insulators is revised within a first principles framework. For the archetypical cases of Bi_2_Se_3_ and Bi_2_Te_3_, we find an overall weak coupling constant of less than 0.15, but with a characteristic energy dependence. Derived electronic self-energies compare favorably with previous angle-resolved photoemission spectroscopy results. The prevailing coupling is carried by optical modes of polar character, which is weakly screened by the metallic surface state and can be reduced by doping into bulk bands. We do not find any indication of a strong coupling to the A_1*g*_ mode or the presence of a Kohn anomaly in the surface phonon spectrum. The weak intrinsic electron-phonon coupling guarantees long-lived quasiparticles at elevated temperatures.

## Introduction

The remarkable feature of 3D topological insulators like Bi_2_Se_3_ is the existence of metallic surface states with a Dirac-like dispersion. These states exhibit a peculiar spin texture, with the spin locked perpendicular to the momentum. Their topological nature guarantees a robustness against scattering on non-magnetic defects, and raised great expectations for applications in spintronics^1, 2^. The topological protection of these surface states, however, holds only within the independent quasiparticle picture, but is not guaranteed in the case of many-body interactions. Among them, inelastic electron-electron scattering^3^ likely has a minor influence on the dispersion and linewidth of the surface states due to the strong reduction of the effective electron-electron interaction by the large dielectric constant of the bulk materials^4, 5^. On the other hand, the detrimental effect of electron-phonon interaction (EPI) increases with increasing temperature and thus could be a limiting factor for applications at elevated temperatures. These many-body effects can be important for a realization of the quantum anomalous Hall effect in magnetic topological insulators at elevated temperatures^6–8^.

The fundamental importance of EPI has stimulated various experimental and theoretical studies to assess its intrinsic strength in the topological surface state. On the experimental side, different techniques were applied to extract information about both the strength of the EPI as well as the energy of the involved phonons, in particular, angle-resolved photoemission spectroscopy (ARPES), which directly probes quasiparticle properties^9–13^, and time-resolved ARPES or pump-probe experiments, which access ultra-fast carrier dynamics^14–16^. More indirect information about EPI was extracted from transport measurements^17, 18^ and optical spectroscopy^19, 20^. Also, helium-atom scattering spectroscopy was used to gain insight on the effect of EPI on surface phonon vibrations^21–23^.

However the reported values for the electron-phonon coupling constant *λ* varied greatly even for the same compound. For Bi_2_Se_3_, various ARPES studies gave values ranging from less than 0.1 up to 3^9–13^. Theoretical studies have focused on the interaction with acoustic surface modes but also gave conflicting results (0.42^24, 25^, 0.25^5^, and 0.01^26^). The latter value seems more reasonable because the experiments^12, 13, 15, 18^ indicate that modes which contribute to the electron-phonon coupling have energies between 8 and 21 meV except for the ARPES measurements^13^ which revealed strong mode couplings in the Dirac cone surface states at an energy of ~3 meV. But it was shown that most probably the mode consists of spin plasmons with an energy of 2.2 meV which were theoretically proposed^27^. Due to a small momentum range of the Dirac cone only long wavelength acoustic phonons can participate in the EPI but their energies are very small. These inconsistencies stress the need to reinvestigate the issue of electron-phonon interaction in topological surface states using *ab initio* calculation methods. They make it possible to accurately analyze all phonon-induced transitions and the role of each phonon mode in the electron-phonon coupling.

First principles approaches to EPI in Dirac states of TI’s are facing two major challenges. Firstly, these topological surface states are rather weakly localized near the surface and are properly represented only on rather thick slabs making the lattice dynamics calculations very demanding. Secondly, the Dirac cone covers only a small momentum range (a few percent of the Brillouin zone) requiring rather fine grids to properly sample both electron and phonon quantities, which was overlooked in ref. 28.

In this work, we theoretically study the EPI in the topological surface states within a fully ab initio approach. We find overall modest coupling strengths, but with a significant dependence on the binding energy which essentially scales with the density of states. The dominant contribution involves optical phonon modes with polar character at small momentum transfer, which is only weakly screened by the presence of the metallic surface state. By doping into bulk states these modes become more screened, which results in a further decrease of the EPI.

## Results and Discussion

### Coupling strength

The effect of EPI is encoded in the self-energy of an electron (hole) quasiparticle. The imaginary part of the self-energy can be expressed as

1 $$\begin{matrix}{\mathrm{Im}{\rm{\Sigma }}}_{{\bf{k}}i}(\varepsilon ) & = & -\pi {\int }_{0}^{\infty }d\omega \{{\alpha }^{2}{F}_{{\bf{k}}i}^{E}(\varepsilon ,\omega )[b(\omega )+f(\omega +\varepsilon )]\\  &  & +{\alpha }^{2}{F}_{{\bf{k}}i}^{A}(\varepsilon ,\omega )[b(\omega )+f(\omega -\varepsilon )]\}\end{matrix}$$

Here *f* and *b* denote Fermi and Bose distribution functions, respectively. The real part $\mathrm{Re}{\rm{\Sigma }}$ can then be calculated from $\mathrm{Im}{\rm{\Sigma }}$ with the help of the Kramers-Kronig relation. The spectral functions entering Eq. (1) express the efficiency to scatter electrons either by absorption or emission of a phonon and are given by

2 $${\alpha }^{2}{F}_{{\bf{k}}i}^{E/A}(\varepsilon ,\omega )=\sum _{{\bf{q}},\nu ,f}\delta (\varepsilon -{\varepsilon }_{{\bf{k}}+{\bf{q}}f}\pm \omega ){|{{\rm{g}}}_{{\bf{k}}+{\bf{q}}{\rm{f}},{\bf{k}}{\rm{i}}}^{{\bf{q}}\nu }|}^{2}\delta (\omega -{\omega }_{{\bf{q}}\nu }).$$

Here the energies of the initial and final electronic states are given by ${\varepsilon }_{{\bf{k}}i}$ and ${\varepsilon }_{{\bf{k}}+{\bf{q}}f}$, respectively, while ${{\rm{g}}}_{{\bf{k}}+{\bf{q}}{\rm{f}},{\bf{k}}{\rm{i}}}^{{\bf{q}}\nu }$ denotes the electron-phonon matrix elements related to a specific phonon mode with momentum *q* and mode index *ν*.

A measure for the coupling strength of an electron in a state with momentum **k** and band index *i* with phonons is given by the dimensionless coupling constant

3 $${\lambda }_{{\bf{k}}i}={\int }_{0}^{{\omega }_{{\rm{\max }}}}\frac{{\alpha }^{2}{F}_{{\bf{k}}i}^{E}(\omega )+{\alpha }^{2}{F}_{{\bf{k}}i}^{A}(\omega )}{\omega }{\rm{d}}\omega ,$$

where ${\alpha }^{2}{F}_{{\bf{k}}i}^{E/A}(\omega )={\alpha }^{2}{F}_{{\bf{k}}i}^{E/A}({\varepsilon }_{{\bf{k}}i},\omega )$. The integration sums over all phonons carrying the coupling up to the maximum phonon frequency ${\omega }_{{\rm{\max }}}$. Density-functional theory provides a coherent framework to calculate all quantities necessary to evaluate Eq. (3). In particular, phonons and electron-phonon coupling matrix elements are obtained with the efficient linear response technique^29^. This approach has been successfully applied to the renormalization of electronic quasiparticles in bulk^30^, at metal surfaces^31^ of spin-orbit split surface states^32, 33^, and of quantum well states of thin films^34, 35^.

**Figure 1:**
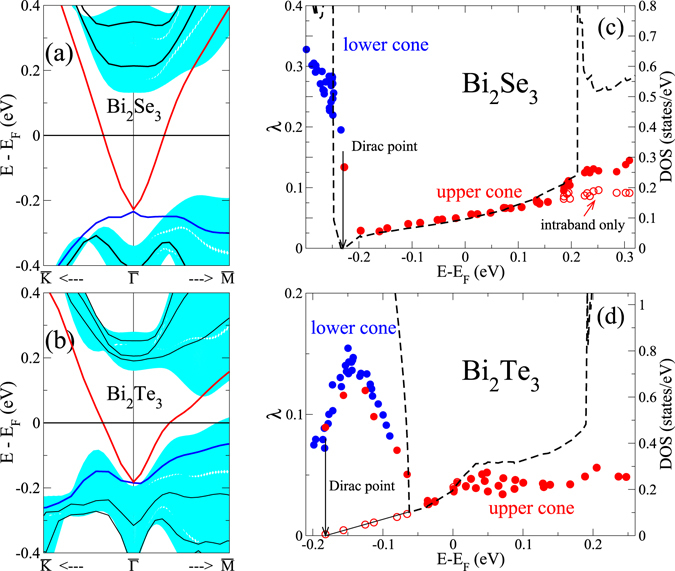
(**a**,**b**) Electronic dispersion of 3 QL Bi_2_Se_3_ (**a**) and 4QL Bi_2_Te_3_ (**b**), respectively. The energy region of surface-projected bulk states is indicated by the shaded area. (**c**,**d**) State-dependent electron-phonon coupling constants for Bi_2_Se_3_ (**c**) and Bi_2_Te_3_ (**d**). *λ* for both upper (red) and lower (blue) part of the Dirac cone is plotted over the energy of the corresponding electronic state. Open (red) circles denote the intraband contribution for the upper part of the cone, i.e. without coupling to the first bulk-like conduction band. The dashed line indicates the density of states (DOS).

In this study, we focus on two prominent examples of 3D TI, Bi_2_Se_3_ and Bi_2_Te_3_, which are known to possess well isolated Dirac cones inside the large bulk bandgap. These compounds are layered materials, built from blocks of quintuple layers (QL), which are bound together via van-der-Waals interaction. Thin films are modeled by slabs consisting of 3QL and 4QL for Bi_2_Se_3_ and Bi_2_Te_3_, respectively. For such thicknesses, the topological surface band is well developed as seen in the surface bandstructures in Fig. 1a,b. Hybridization gaps of 6.2 meV and 2.5 meV occur at the Dirac point of Bi_2_Se_3_ and Bi_2_Te_3_, respectively, which are small with regard to the bulk band gaps of these materials, consistent with previous publications^36–39^. The small hybridization gaps demonstrate that the Dirac states on opposite surfaces of the slab are sufficiently separated. These small residual gaps do not affect the following discussion. For both compounds, only the upper part of the cone exhibits a linear dispersion, while the lower one remains rather flat. To mimic a typical experimental situation, we considered a slight n-type doping for Bi_2_Se_3_, which puts the Fermi level inside the upper part of the Dirac cone, but well below the first bulk-like conduction band.

The electron-phonon coupling constants *λ*_**k***i*_ of Bi_2_Se_3_ are plotted over the band energies in Fig. 1c. For the upper part of the Dirac cone, *λ*_**k***i*_ exhibits a monotonic increase with the energy distance from the Dirac point, following essentially the linear increase of the density of states (DOS) expected for the 2D Dirac dispersion. Most importantly, its value does not exceed 0.15. The fact that the values of *λ*_**k***i*_ fall on a single line highlights the isotropy of the coupling in the upper part of the cone. For states at higher energies (*E* − *E*_*F*_ > 0.2 eV) an additional decay into bulk-like conduction bands becomes possible, which adds to the intraband contribution and enhances the coupling constant by 40%. Additional calculations verified that these results are robust with respect to variations in the film geometry, e.g. slab thickness or size of the vacuum (for more details, see Supplementary Information).

The coupling strength related to an electronic state with energy *ε* is determined essentially by two factors: the number of final electronic states available with energies $\varepsilon \pm {\omega }_{ph}$, where ${\omega }_{ph}$ is the frequency of the absorbed/emitted phonon, and the probability of scattering between the two electronic states given by the EPI matrix elements. The observation that *λ*_**k***i*_ is proportional to the DOS suggests that the energy dependence is determined by the first factor and that the matrix elements are largely independent of the energy of the electronic state. They possess, however, an pronounced dependence on the momentum transfer, as will be discussed below.

For Bi_2_Te_3_, the couplings are more than a factor of 2 smaller than for Bi_2_Se_3_ (Fig. 1d). At the same time, the DOS and thus the number of available states is slightly enhanced. Therefore, the reduced coupling indicates that the EPI matrix elements for Bi_2_Te_3_ are significantly smaller than for Bi_2_Se_3_. The linear dependence of *λ*_**k***i*_ on the energy is less obvious, because the lower and upper part of the Dirac cone overlap in energy (see Fig. 1b), which enables significant interband coupling below *E*_*F*_. However, when only intraband scattering is considered the expected linear behavior up to $\approx$150 meV above the Dirac point is recovered. For higher energies the electronic dispersion starts to deviate from the linear behavior and develops a hexagonal warping. Simultaneously we find a pronounced anisotropy in the coupling constant, which manifests itself by the scattering of *λ* values for a given energy (Fig. 1d). For a fixed energy, the coupling becomes largest along the $\overline{{\rm{\Gamma }}{\rm{K}}}$ direction, and minimal along $\overline{{\rm{\Gamma }}{\rm{M}}}$. This is in line with ARPES observations of anisotropic lifetime broadenings of the topological surface state, being larger along $\overline{{\rm{\Gamma }}{\rm{K}}}$ than along $\overline{{\rm{\Gamma }}{\rm{M}}}$ ^40^.

For the quite flat lower part of the cone, on the contrary, values reach up to *λ* = 0.35 for Bi_2_Se_3_, which correlates with the increase of the DOS as compared to the upper part of the cone. For Bi_2_Te_3_, the coupling is again more than 50% smaller. Because of the flat dispersion, this part of the Dirac cone is experimentally difficult to access, and we will restrict the following analysis to the upper part only.

### Spectral decomposition

**Figure 2:**
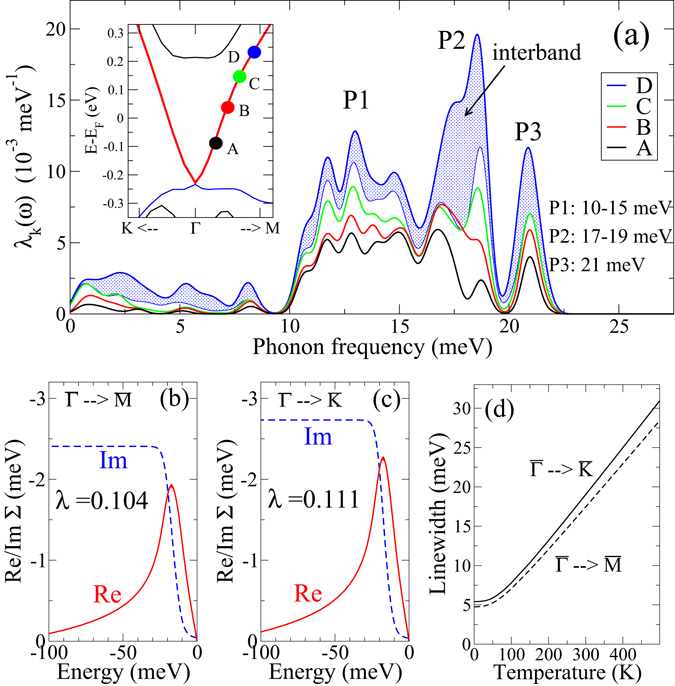
(**a**) Spectral representation of the coupling for four different states (A–D) of the upper cone surface state along the $\overline{{\rm{\Gamma }}{\rm{M}}}$ direction for the 3QL slab of Bi_2_Se_3_. The position of the states is indicated in the inset. P1–P3 denote the energies of the three dominant contributions. The shaded area represents the interband contribution for state D. (**b**,**c**) Real and imaginary part of the self-energy for two states with energies *E* − *E*_*F*_ = 200 meV along (**b**) $\overline{{\rm{\Gamma }}{\rm{M}}}$ and (**c**) $\overline{{\rm{\Gamma }}{\rm{K}}}$, evaluated for *T* = 20 K. (**d**) Temperature dependence of the electronic linewidth (full width at half maximum) for the two states of (**b**,**c**).

Insight into the type of phonon modes which carry the coupling can be gained from the spectral decomposition of the coupling constant, ${\lambda }_{{\bf{k}}i}=\int {\lambda }_{{\bf{k}}i}(\omega )d\omega$, with ${\lambda }_{{\bf{k}}i}(\omega )=({\alpha }^{2}{F}_{{\bf{k}}i}^{E}(\omega )+{\alpha }^{2}{F}_{{\bf{k}}i}^{A}(\omega ))/\omega$. It gives the contribution of modes with frequencies $\omega$ to the total coupling constant. Figure 2a shows typical spectra obtained for states of the upper part of the cone. Four states along $\overline{{\rm{\Gamma }}{\rm{M}}}$ with different energies were chosen (denoted by A to D in the inset of Fig. 2a). All spectra are dominated by optical modes with $\omega  > 10$ meV, while the contribution of low-energy modes including acoustic ones is marginal. This contrasts with previous theoretical studies of electron-phonon coupling based on continuum phonon models, which suggested large contributions of 0.25 up to 0.84 from the acoustic modes alone^5, 25^. The shape of all spectra is very similar, but their magnitude increases along the sequence A → B → C → D, which reflects the growth of the coupling strength *λ*_**k***i*_ with increasing energy of the electronic state. For the state with the highest energy, in addition to intra-cone scattering, also decay via states of the first bulk-like conduction band is energetically possible. This interband channel mainly involves optical modes with frequencies around 18 meV, but also some enhanced contribution from modes below 10 meV can be inferred from its spectrum.

The spectral decomposition determines the shape and size of the electronic self-energy Eq. (1), which is accessible via ARPES measurements. Chen *et al*. were able to extract the self-energy of states lying about 350 meV above the Dirac point for both $\overline{{\rm{\Gamma }}{\rm{M}}}$ and $\overline{{\rm{\Gamma }}{\rm{K}}}$ directions^12^. Figure 2b,c show calculated self-energies for the states of similar energy position in the upper part of the Dirac cone. Peaks in the real part occur in both cases at $\approx$17 meV in very good agreement with 18 meV found by Chen *et al*. As in the experiment, we find a small anisotropy with a slightly larger self-energy for the $\overline{{\rm{\Gamma }}{\rm{K}}}$ direction. Also the experimental peak height of Re${\rm{\Sigma }}$ of $\approx$2.5 meV for the $\overline{{\rm{\Gamma }}{\rm{K}}}$ direction is well matched by our calculation. Despite these quantitative agreements, our value of *λ* = 0.111 is 30% smaller than the value of *λ* = 0.17 deduced by Chen *et al*. from the slope of Re${\rm{\Sigma }}(E)$. This discrepancy can be traced back to the fact that the calculated Re${\rm{\Sigma }}(E)$ has a significant curvature between −20 meV and 0 meV because the contribution from low-frequency phonons is very small. Such a curvature cannot be resolved in experiment. Because *λ* is related to the slope of Re${\rm{\Sigma }}(E)$ right at *E* = *E*_*F*_, this demonstrates the difficulty of extracting accurate values for *λ* from ARPES experiments. The EPI induced electronic linewidth ${{\rm{\Gamma }}}_{{\bf{k}}i}$ can be directly obtained from the imaginary part of the self-energies via ${{\rm{\Gamma }}}_{{\bf{k}}i}=-2\mathrm{Im}{\rm{\Sigma }}({\varepsilon }_{{\bf{k}}i})$. The *T*-dependence of ${\rm{\Gamma }}$ for the two states considered gives typical linewidths of 20 meV at room temperature (Fig. 2d). Thus quasiparticles are well defined at elevated temperatures.

### Mode analysis

**Figure 3:**
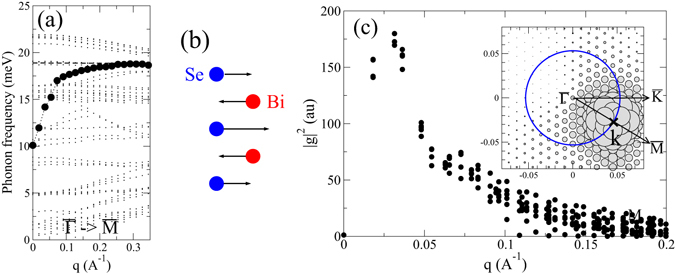
(**a**) Phonon dispersion of the 3QL slab of Bi_2_Se_3_ in the vicinity of $\overline{{\rm{\Gamma }}}$ along the $\overline{{\rm{\Gamma }}{\rm{M}}}$ direction. Circles highlight the longitudinal branch of the in-plane polar-type mode. (**b**) Displacement pattern of the same mode. (**c**) Dependence of the squared electron-phonon matrix elements, |*g*|^2^, on the modulus of the momentum transfer *q*, for a fixed initial state along $\overline{{\rm{\Gamma }}{\rm{M}}}$, as carried by the in-plane polar-type vibration. The inset shows the full 2D momentum dependence of $|{g}_{{\bf{k}}+{\bf{q}},{\bf{k}}}{|}^{2}$ for an initial state along with momentum **k** (position is indicated by the cross) as function of the final momentum **k** + **q**. The size of the filled circles is proportional to *g* ^2^. The blue circle indicates states with the same energy as the initial state.

To gain further insight into the microscopic nature of the EPI, we identified the type of optical modes actually involved in the coupling. Significant contributions come from modes with a polar-type character. The dominant coupling has its origin in a longitudinal in-plane polarized mode. Here Bi and Se vibrate in anti-phase, as depicted in Fig. 3b. As seen from the phonon dispersion along the $\overline{{\rm{\Gamma }}{\rm{M}}}$ direction in Fig. 3a, this branch possesses a steep dispersion in the vicinity of $\overline{{\rm{\Gamma }}}$, starting from 10 meV at $\overline{{\rm{\Gamma }}}$ and quickly approaching 19 meV. This behavior contrasts with the rather weak dispersion of other branches, and can be understood on the following grounds.

In the semiconducting bulk, this mode is infrared active (IR) and shows a large LO/TO splitting^41^ (The bulk TO frequency was observed at about 8.1 meV^41, 42^ at room temperature. In our calculation, this modes lies at a slightly higher energy of 9.8 meV, see Supplementary Information). For the slab, due to the presence of the metallic surface state, the macroscopic electric field created by the polar mode is screened for small wavelength, and the LO/TO splitting is suppressed at the Brillouin zone center. The relevant screening length is determined by the size of the Dirac cone and thus is of the order of 0.1 Å^−1^. For larger momenta the longitudinal branch quickly approaches the LO frequency of 19 meV.

It is well known that IR active modes in polar semiconductors promote singular EPI, where the EPI matrix elements diverge as 1/*q* when the phonon momentum *q* approaches zero^43^ (polar-optic coupling or Fröhlich interaction). Because of the weak screening a tendency to diverge is also inherent for matrix elements related to the Dirac state. Figure 3c shows the dependence of |*g*(*q*)|^2^ for a fixed initial state (state B in the inset of Fig. 2a) as a function of the momentum transfer *q*. |*g*(*q*)|^2^ strongly increases for *q* → 0 down to $q\approx 0.03$ Å^−1^, before the screening suppresses it for very small *q*.

The small scattering of values as a function of *q* in Fig. 3c also indicates a rather isotropic dependence of $|{g}_{{\bf{k}}+{\bf{q}},{\bf{k}}}^{{\bf{q}}}|$ on the momentum transfer **q** for intra-cone couplings. This isotropy is also seen in the 2D representation shown in the inset of Fig. 3c. In addition, a suppression of the coupling for backscattering (${\bf{k}}+{\bf{q}}\approx -{\bf{k}}$) is clearly visible. It has its origin in the peculiar spin structure of the surface state, where the spins on opposite sides of the Dirac cone are locked in an antiparallel configuration.

This strongly dispersive polar-type mode is responsible for the broad feature denoted by P1 in Fig. 2a. It also contributes partly to the peak P2, together with a non-dispersive *c*-polarized phonon branch at 19 meV. Finally, the peak P3 at about 21 meV originates from a phonon mode, where Bi and Se vibrate against each other along the *c* direction, and is associated to the high-frequency IR *A*_*u*_ mode of the bulk. Their EPI matrix elements, in contrast to the case of the in-plane polar-type mode, are not enhanced for small *q* because the polarization is vertical to the surface and thus purely transversal.

### Doping

**Figure 4:**
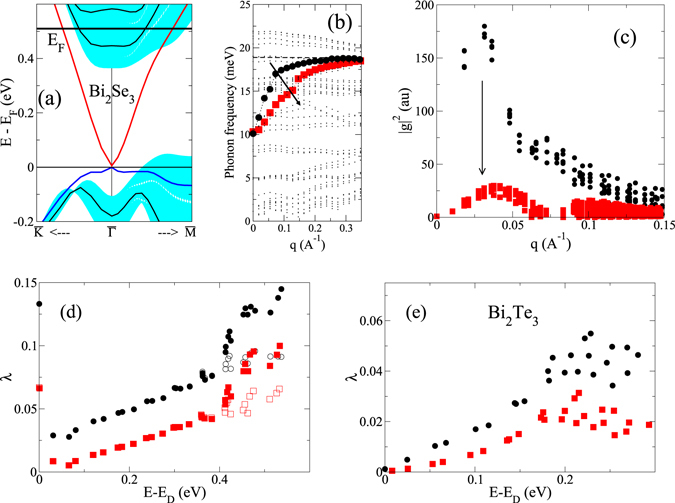
(**a**–**d**) Changes of EPI under doping for Bi_2_Se_3_ (3QL). (**a**) Bandstructure indicating position of Fermi level. (**b**) Phonon dispersion along the $\overline{{\rm{\Gamma }}{\rm{M}}}$ direction. Large, filled squares highlight the in-plane polar-type branch. The dispersion for low doping (see Fig. 1) is shown by the black circles for comparison. The arrow indicates the enhanced screening effect on this mode. (**c**) Squared electron-phonon matrix elements, |*g*|^2^, as function of the momentum transfer *q*. Red squares correspond to heavy doping, black circles to the slightly doped case, respectively. (**d**) Coupling constants for the upper part of the Dirac cone comparing the two dopings (read squares: large doping; black circles: low doping). (**e**) Coupling constants for a 4QL slab of Bi_2_Te_3_ comparing undoped (black circles) and the heavily doped case (red squares).

The finding that an important part of the coupling is carried by a polar-type mode suggests that the quasiparticle renormalization could be dependent on the degree of doping. We have therefore performed similar calculation for different doping levels by variation of the electron content in the slabs. As long as the doping is small such that the Fermi level remains inside the bulk gap, the coupling strength of individual states, i.e. *λ*_**k***i*_, stays practically the same. The behavior changes when a sufficient amount of electrons is added to populate the first bulk-like conduction band. Figure 4a–d show results for the 3QL slab of Bi_2_Se_3_ with 0.12 electrons added per unit cell. The dispersion of the polar-type mode becomes flatter (Fig. 4b) indicating that the screening now extends over a larger momentum range. Simultaneously, the related EPI matrix elements are greatly reduced (Fig. 4c). As a consequence, the coupling constant *λ* is reduced for all states in the topological surface state (Fig. 4d). Populating the conduction bands thus has two opposite effects: (i) for states at the Fermi level, the coupling gets enhanced because additional decay into bulk states becomes available, and (ii) the additional screening reduces the coupling via polar-type modes. For Bi_2_Se_3_ these two effects are of the same order of magnitude (Fig. 4d).

The same trend is also found for Bi_2_Te_3_. Here we considered a doping with 0.32 electrons per unit cell of the 4QL slab, by which the first three conduction bands become partly populated (see Fig. 1b). As a result of the enhanced screening, the coupling constants are reduced by almost 50% (Fig. 4e) for states in the upper part of the Dirac cone.

## Discussion

The present calculations clearly show that the EPI in the topological surface state is of the order of *λ* = 0.1 and thus weak enough to guarantee well defined and long-lived quasiparticles at room temperature. Our finding of a doping dependence of the coupling may partly explain the variation in experimental values, because the degree of doping into bulk states depends sensitively on the sample preparation. Furthermore, the energy dependence of *λ* adds another parameter which differs among various measurements.

The relevance of the polar mode and its related strong polar EPI has been stressed before in the context of transport and optical spectroscopy studies. Surface transport in thin films of Bi_2_Se_3_ showed an activated behavior attributed to a strong EPI via an optical phonon of about 8 meV^18^, which matches the frequency of the polar-optic mode in the bulk. Reflectivity measurements on thin films of Bi_2_Se_3_ also suggested that the same polar mode underlies the relaxation mechanism for hot electrons^44^, and an interference between this mode and a Dirac plasmon has been observed by infrared absorption techniques^45^.

Furthermore, our results have implications for the interpretation of a variety of previous experiments. In a recent time-resolved ARPES measurement on Bi_2_Se_3_, a coherent excitation of the A_1*g*_ phonon mode (at 8.48 meV) was achieved, which modulated the band dispersion of the surface state^15^. This provided the first experimental evidence of a direct coupling between the A_1*g*_ mode and the topological surface state. In the present calculation, we also find a contribution of the A_1*g*_ mode to the coupling, but it is quite small. In the 3QL case, the small peak at $\approx$8 meV in the spectra of Fig. 2a results from such a coupling to the A_1*g*_ mode.

Helium-atom scattering experiments on both Bi_2_Se_3_ and Bi_2_Te_3_ suggested the presence of strong Kohn anomalies in low-frequency surface phonon branches (below 8 meV) and were interpreted as signatures of a strong electron-phonon coupling to the metallic surface electronic bands^21–23^. The Kohn anomaly was tentatively assigned to the branch connected to the A_1*g*_ mode, and estimates for branch-specific coupling constants gave large values of 0.43 and 1.44 for Bi_2_Se_3_ and Bi_2_Te_3_, respectively. Our slab calculations for thin films of 3QL and 4QL contain surface localized modes, and together with the well represented Dirac state they are well suited to capture couplings between the metallic surface state and surface phonons. Yet, our results do neither support large coupling constants nor the presence of Kohn anomalies. For example, the suggested softening with increasing momentum of a mode starting at $\overline{{\rm{\Gamma }}}$ at 7–8 meV is clearly absent in the dispersion of the 3QL Bi_2_Se_3_ slab (Fig. 3). It is thus unlikely that the HAS results do reflect a strong EPI of the surface state. Instead, they may be connected to a larger EPI in the doped bulk material. A recent first principles study suggested that doping into the conduction bands of bulk Bi_2_Se_3_ by Cu intercalation activates a peculiar form of EPI^46^, which could be responsible for the superconductivity observed in Cu doped Bi_2_Se_3_^47^.

Raman measurements on Bi_2_Se_3_ thin films, capped with a MgFe_2_ layer for protection against oxidation, detected a mode at about 31 meV (H-mode), which was assigned to a surface vibration involving the outmost Se layer^19^. Its Fano lineshape suggested a significant EPI which became larger for thinner films^20^. Our calculations for free-standing slabs show that the uncovered surface of Bi_2_Se_3_ does not possess surface modes with frequencies above the maximum bulk frequency of 22 meV, which is in line with previous calculations for 1QL and 2QL films^48^. Thus we can exclude that the observed H-mode is an intrinsic surface mode, but it may be instead a consequence of the capping of the sample surface with the MgFe_2_ layer.

## Conclusions

Our first principles calculations show that the intrinsic electron-phonon coupling in the topological surface state of Bi_2_Se_3_ and Bi_2_Te_3_ is of the order of 0.1 and thus small enough to support well defined quasiparticles even at room temperature. Shape and magnitude of derived electronic self-energies compare favorably with ARPES measurements. We found a characteristic linear dependence of the coupling strength on the quasiparticle energy as well as a reduction induced by doping into bulk states. Both properties could partly explain why previous experiments lead to a larger range of values for *λ*, but results larger than 0.2 are not compatible with intrinsic EPI. A microscopic analysis revealed that the coupling in the Dirac cone is dominated by optical modes, with an important contribution coming from a weakly screened polar-type coupling. For the investigated ultra-thin films, our results do neither support the presence of Kohn-type anomalies nor the existence of a strong coupling mode with frequency well above the bulk phonon spectrum, as was inferred from previous Helium-atom or Raman spectroscopy studies, respectively.

The present findings can also be important for realizations of the quantum anomalous Hall effect (QAHE), which relies on sufficiently large band gaps in the Dirac cone introduced either by magnetic dopants or by surface interactions of TI films with magnetic semiconductors^6–8^. The small EPI implies that gap sizes are not severely reduced by phonon-induced many-body effects, which supports the quest for devices which exhibit the QAHE at significantly higher temperatures than can be achieved nowadays.

## Methods

Electronic structure, lattice vibrations and electron-phonon coupling matrix elements were calculated within density-functional theory and density-functional perturbation theory, respectively. We employed the mixed-basis pseudopotential approach, where the interaction of electrons with the ion cores is expressed in terms of norm-conserving pseudopotentials, and a combination of plane waves and local orbitals is used to represent the valence states^49–51^. By using *s* and $p$-type local functions at the Bi, Se, and Te sites, the cutoff energy for the plane waves could be reduced to 10 Ry without loss of accuracy. Only due to the small basis-set size, sufficiently thick slabs became tractable. The PBE parameterization of the generalized-gradient approximation was used for the exchange-correlation functional^52^. Spin-orbit coupling is consistently taken into account for all calculated quantities^53^. To deal with the small momentum range covered by the Dirac cone, we employed inhomogeneous *k*-point grids, where a very dense 96 × 96 hexagonal grid near the zone center was combined with a coarse 12 × 12 mesh for sampling the remainder of the surface Brillouin zone. Doping was simulated by adding a small amount of electrons, whose charge is compensated by a homogeneous background charge.

## Electronic supplementary material

Supplementary Information
