# Supplementary material for: Electron-phonon coupling in topological surface states: The role of polar optical modes
Source: Sci Rep. 2017 Apr 24;7:1095. doi: 10.1038/s41598-017-01128-1 (PMC5430646; doi:10.1038/s41598-017-01128-1)
Supplement: Supplementary file 1 — Supplementary Information [file 41598_2017_1128_MOESM1_ESM.pdf]

Supplementary Information

Electron-phonon coupling in topological surface states: The role of polar optical modes

Rolf Heid,[1] Irina Yu. Sklyadneva,[2] Evgueni V. Chulkov[2,3,4,5]

[1]Institut für Festkörperphysik, Karlsruher Institut für Technologie, D-76021 Karlsruhe, Germany

[2]Donostia International Physics Center (DIPC), 20018 San Sebastián/Donostia, Basque Country, Spain

[3]St. Petersburg State University, 199034, St. Petersburg, Russian Federation

[4]Departamento de Fsica de Materiales, Facultad de Ciencias Qumicas, UPV/EHU, Apdo. 1072, 20080 San Sebastián/Donostia, Basque Country, Spain

[5]Centro de Fsica de Materiales CFM - Materials Physics Center MPC, Centro Mixto CSIC-UPV/EHU, 20018 San Sebastián/Donostia, Basque Country, Spain

Bulk lattice structure and phonons

The lattice structures of Bi_2Se_3 and Bi_2Te_3 consist of quintuple layers (QL) in the sequence Se/Te - Bi - Se/Te - Bi - Se/Te, separated by van der Waals (vdW) gaps. It is well known, that if one performs a full structural optimization, LDA/GGA functionals do not reproduce QL separation properly. We have therefore used experimental lattice constants taken from [1] and performed a relaxation of the internal structural parameters. Obtained values are collected in Table S1.

Compound	a_h(Å)	c_h(Å)	z(Bi)	z(Se/Te)	
Bi_2Se_3	4.1375	28.640	0.399	0.206	exp
			0.4012	0.2094	relaxed
Bi_2Te_3	4.3835	30.487	0.400	0.212	exp
			0.4004	0.2084	relaxed

TABLE S1: Bulk structural parameters for Bi_2Se_3 and Bi_2Te_3. Experimental values are taken from [1].

Frequencies of the Γ phonons calculated for these relaxed geometries are compared in Table S2 with previous theoretical and experimental studies.

	A^1_{1g}	A^2_{1g}	E^1_g	E^2_g	A^1_{1u}	A^2_{1u}	E^1_u	E^2_u	Reference
Bi_2Se_3	8.9	21.5	5.0	16.4	17.0	20.2	9.8	15.9	present work
	8.5	21.1	5.7	16.2					Chis *et al.* [2]
	9.36	21.2	5.15	17.0	17.0	20.0	9.96	16.2	Cheng *et al.* [3]
	9.0	21.9		16.3					Chis *et al.* [2], 300 K
	8.9	21.6		16.3			8.1	16.0	Richter *et al.* [4], 300 K
							7.6	16.6	Richter *et al.* [4], 15 K
	9.1	21.76	4.84	16.46	16.06	19.85	8.44	15.5	Gnezdilov *et al.* [5], 3 K
Bi_2Te_3	7.8	16.4	4.9	12.6	12.05	15.1	7.6	11.6	present work
	6.7	15.8	4.4	11.9	11.8	14.7	6.0	11.3	Cheng *et al.* [3]
	7.7	16.6	4.4	12.6	12.1	15.2	7.0	11.5	Huang *et al.* [6]
	7.8	16.6		12.8	11.7	14.9	6.2	11.8	Richter *et al.* [4], 300 K
					10.9	14.1	6.0	12.2	Richter *et al.* [4], 15 K
	7.7	16.5	4.3	12.6					Shalil *et al.* [7], 300 K

TABLE S2: Bulk phonon frequencies (in meV) at Γ from various theoretical and experimental works. The latter are indicated by the respective measurement temperature.

Slab geometries and convergence tests

Periodic slabs were set up taking the relaxed QL blocks from bulk separated by the bulk vdW gap without further relaxation. For Bi_2Se_3, a slab consisting of 3QL was sufficient to reduce the gap at the Dirac point below 7 meV, while for Bi_2Te_3 a 4 QL slab was needed. Large vacua of 11.0 Å and 12.3 Å separating the slabs perpendicular to the surface were chosen for Bi_2Se_3 and Bi_2Te_3, respectively.

To test the robustness of our results for the electron-phonon coupling, we performed several calculations for Bi_2Se_3 with varied geometries, using the 3QL slab as reference:

(1) Vacuum size: increase of vacuum thickness from 11.0 Å to 31.7 Å.

(2) Slab thickness: addition of another QL (3QL → 4QL)

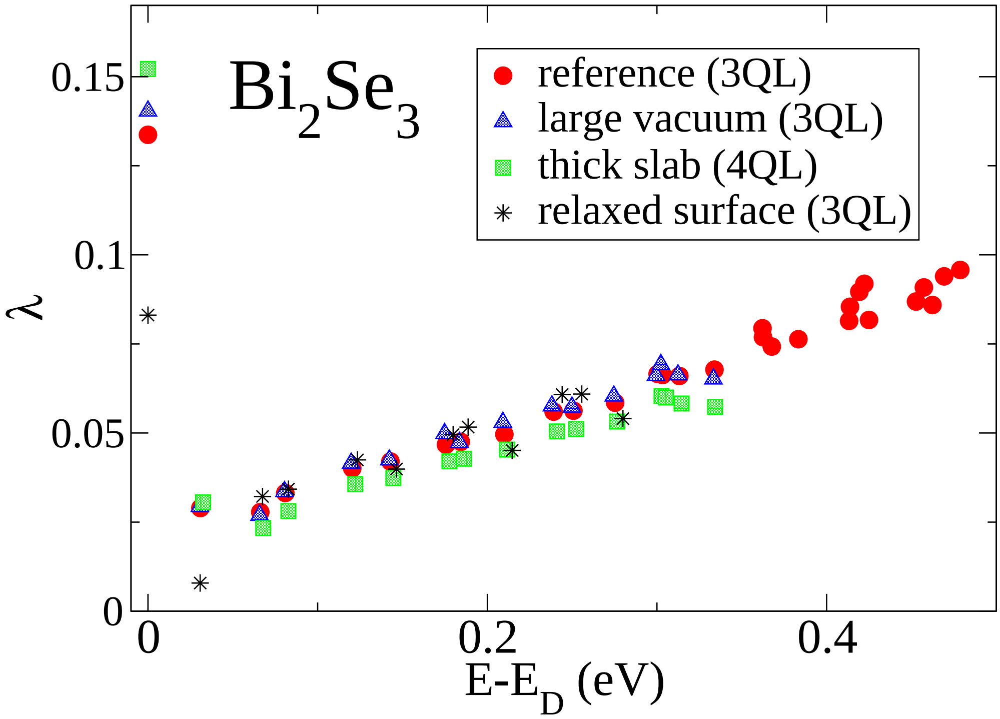

Figure S1: Coupling constants for Bi_2Se_3 for different geometries (see text). The reference geometry denotes the unrelaxed 3QL slab with 11.2Å vacuum. All cases refer to small doping, where the Fermi energy lies in the upper part of the Dirac cone (Fig. 1a of main article).

(3) Surface relaxation: we allowed the 4 outer layers on each side of the 3QL slab to relax freely. We obtained a general small outward relaxation, with changes in the the first 4 interlayer distances of 0.3%, 2.1%, 1.9%, and 2.0%. This small magnitude agrees with experimental findings, although details of the relaxation are still controversial.[8–10]

For the three different test geometries results for the coupling constants in the upper part of the Dirac cone are collected in Fig. S1. They are almost identical with the 3QL reference slab and demonstrate that our results are well converged with respect to the slab geometry.

---

[1] R. Wyckoff, *Crystal structures*, Crystal Structures No. Bd. 2 (Interscience Publishers, 1964).

[2] V. Chis, I. Y. Sklyadneva, K. A. Kokh, V. A. Volodin, O. E. Tereshchenko, and E. V. Chulkov, Phys. Rev. B **86**, 174304 (2012).

[3] W. Cheng and S.-F. Ren, Phys. Rev. B **83**, 094301 (2011).

[4] W. Richter and C. R. Becker, physica status solidi (b) **84**, 619 (1977).

[5] V. Gnezdilov, Y. G. Pashkevich, H. Berger, E. Pomjakushina, K. Conder, and P. Lemmens, Phys. Rev. B **84**, 195118 (2011).

[6] G. Q. Huang, EPL (Europhysics Letters) **100**, 17001 (2012).

[7] K. M. F. Shahil, M. Z. Hossain, D. Teweldebrhan, and A. A. Balandin, Applied Physics Letters **96**, 153103 (2010).

[8] D. D. dos Reis, L. Barreto, M. Bianchi, G. A. S. Ribeiro, E. A. Soares, W. S. o. e. Silva, V. E. de Carvalho, J. Rawle, M. Hoesch, C. Nicklin, W. P. Fernandes, J. Mi, B. B. Iversen, and P. Hofmann, Phys. Rev. B **88**, 041404 (2013).

[9] S. Roy, H. L. Meyerheim, A. Ernst, K. Mohseni, C. Tusche, M. G. Vergniory, T. V. Menshchikova, M. M. Otrokov, A. G. Ryabishchenkova, Z. S. Aliev, M. B. Babanly, K. A. Kokh, O. E. Tereshchenko, E. V. Chulkov, J. Schneider, and J. Kirschner, Phys. Rev. Lett. **113**, 116802 (2014).

[10] M. V. Kuznetsov, L. V. Yashina, J. Sánchez-Barriga, I. I. Ogorodnikov, A. S. Vorokh, A. A. Volykhov, R. J. Koch, V. S. Neudachina, M. E. Tamm, A. P. Sirotina, A. Y. Varykhalov, G. Springholz, G. Bauer, J. D. Riley, and O. Rader, Phys. Rev. B **91**, 085402 (2015).
